# Assessing local adaptation vs. plasticity under different resource conditions in seedlings of a dominant boreal tree species

**DOI:** 10.1093/aobpla/ply004

**Published:** 2018-01-19

**Authors:** Anastasia E Sniderhan, Gordon G McNickle, Jennifer L Baltzer

**Affiliations:** 1 Department of Geography and Environmental Studies, Wilfrid Laurier University, Waterloo, ON, Canada; 2 Department of Biology, Wilfrid Laurier University, Waterloo, ON, Canada; 3 Department of Botany and Plant Pathology, Purdue University, West Lafayette, IN, USA; 4 Purdue Center for Plant Biology, Purdue University, West Lafayette, IN, USA

**Keywords:** Boreal forest, climate change, common garden, functional traits, resilience, resource availability

## Abstract

Under changing climate conditions, understanding local adaptation of plants is crucial to predicting the resilience of ecosystems. We selected black spruce (*Picea mariana*), the most dominant tree species in the North American boreal forest, in order to evaluate local adaptation vs. plasticity across regions experiencing some of the most extreme climate warming globally. Seeds from three provenances across the latitudinal extent of this species in northwestern Canada were planted in a common garden study in growth chambers. Two levels of two resource conditions were applied (low/high nutrient and ambient/elevated CO_2_) in a fully factorial design and we measured physiological traits, allocational traits, growth and survival. We found significant differences in height, root length and biomass among populations, with southern populations producing the largest seedlings. However, we did not detect meaningful significant differences among nutrient or CO_2_ treatments in any traits measured, and there were no consistent population-level differences in physiological traits or allocation patterns. We found that there was greater mortality after simulated winter in the high nutrient treatment, which may reflect an important shift in seedling growth strategies under increased resource availability. Our study provides important insight into how this dominant boreal tree species might respond to the changing climate conditions predicted in this region.

## Teaser

In this study, we used a common garden to determine if there is differentiation among black spruce seedlings from populations across 15° of latitude in western Canada. Additionally, we tested whether seedlings exhibit trait plasticity in response to increased resource availability. Our results clearly indicated that southern populations grow larger, faster and longer than their northern counterparts. However, there were no differences in allocation patterns or physiological traits among populations and little evidence of plasticity. This study on population-level differentiation and trait plasticity in black spruce contributes to better understanding the resilience of this species under a changing climate.

## Introduction

Changes in global climate patterns over the past century have led to shifts in climate envelopes, that is, the set of climatological characteristics that delineate the extent of a species’ range (Walther *et al.* 2002). These shifts in climate present organisms with a limited set of options: migrate at the same rate as the climate is shifting, adapt to the new conditions through genetic change or tolerate the new conditions through plasticity in their functional traits (Aitken *et al.* 2008). Trees are particularly vulnerable to changes in climatic conditions that influence survival and reproduction, since most trees are long-lived and their migration or genetic adaption may not keep pace with changing climate conditions (Jump and Peñuelas 2005).

The capacity of individual trees to exhibit trait plasticity (i.e. the ability to express different phenotypes under different environments; e.g. Sultan 1995) is an important strategy for trees to adapt to environmental variability, and thus to acclimate to climate change. As a response to the rapidly changing climate, plasticity is a means of acclimating to the effects of these changes (Sultan 2000). Alternatively, trees with fixed traits that are no longer compatible with current conditions may be fated to mortality, either through environmental stress or interspecific competition to better-adapted species (Aitken *et al.* 2008; Allen *et al.* 2015). However, the degree of plasticity vs. fixed traits is not necessarily consistent within a given tree species. There have been observations of variability in the presence or degree of trait plasticity among populations (e.g. Benito Garzón *et al.* 2011). For example, a study on *Pinus sylvestris* demonstrated latitudinal differentiation in plasticity of the timing of growth cessation under variable climate conditions (Savolainen *et al.* 2004)—likely as a result of the high cost of maintaining plasticity in the face of resource limitations at some sites (DeWitt *et al.* 1998). These cost-benefit trade-offs in plants reflect the fast–slow trait spectrum; plants with slow trait strategies maintain low rates of resource acquisition and expenditure, whereas those with fast trait strategies require high rates of resource uptake and use (Reich 2014). There is evidence that this fast–slow continuum corresponds with the degree of plasticity that species can maintain; individuals from populations adapted to higher resource conditions show greater plasticity in their traits than those from resource-limited populations, which take a slow and steady approach that does not support plasticity. This pattern is seen in studies along elevational gradients that have found that plasticity of some tree seedlings is lower at higher altitudes (e.g. Green 2005; Vitasse *et al.* 2013), but the fast–slow spectrum has not been investigated across latitudinal ranges.

The boreal region of northwestern North America has experienced some of the world’s most dramatic changes in climate (Chapin *et al.* 2005). Since the late 1800s, the mean annual temperature in this region has increased by up to 2.5 °C (Hartmann *et al.* 2013), and it is predicted to warm up to an additional 4 °C by the end of the 21st century (Flato and Boer 2001; Scinocca *et al.* 2008). Despite the possible negative impacts described above, warming can provide favourable growth conditions for trees through greater plant-available nutrients (due to increased soil microbial activity and in some areas greater permafrost thaw) as well as increased atmospheric CO_2_ concentrations which can provide a fertilization effect (e.g. Bonan and Shugart 1989; Keeling *et al.* 1996).

**Figure 1.**
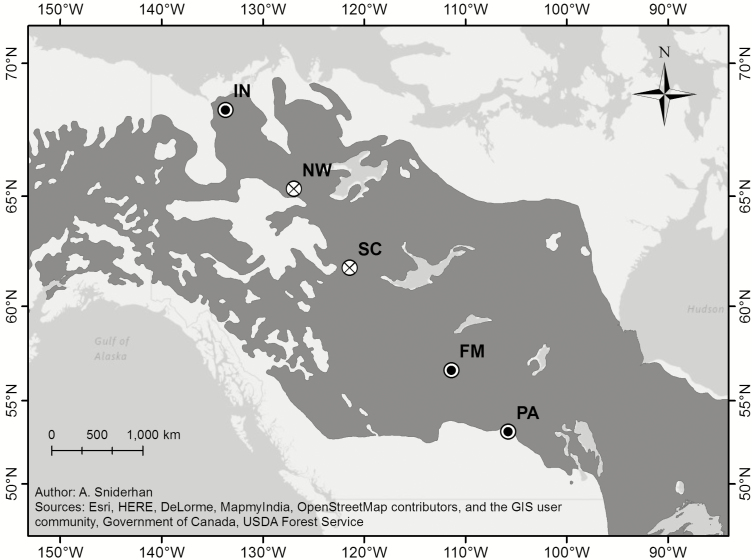
Seed sources for the experiment. IN—Inuvik; NW—Norman Wells; SC—Scotty Creek; FM—Fort McMurray; PA—Prince Albert. Sites marked over with a × indicate populations that suffered extensive mortality before the end of the experiment and could not be included in the analyses. Dark grey shading shows the distribution of black spruce.

Black spruce (*Picea mariana*) is the most dominant tree species in northwestern Canada, and thus an important subject to study in order to understand widespread boreal forest dynamics. Overall, we aim to address three key questions about this species in northwestern Canada: (i) Do populations demonstrate genetic differentiation? (ii) Is genetic differentiation consistent among different types of plant traits? (iii) Is there evidence for differences in plasticity across populations? In this study, we performed a common garden study using black spruce seeds from five sites across a 2250 km transect—effectively capturing the latitudinal extent of this species in northwestern Canada (Fig. 1). By comparing a range of plant traits (physiological, growth and allocational; **see Supporting Information—Table S1** for a complete list), we can assess similarities and differences among populations permitting us to identify whether there is local adaption or plasticity in traits of this widespread species. In addition, our study investigated traits under four conditions of resource availability.

We expect the results of this study to have clear latitudinal patterns. This follows the basic understanding of patterns in plant economics resulting from trade-offs between productivity and stress tolerance in plants—particularly with respect to ‘slow’ and ‘fast’ trait strategies (Reich 2014). For the northern trees in our study, which must withstand very harsh conditions at the extreme limit of the species, we expect a slow trait strategy. A fast trait strategy would be maladaptive in resource poor environments, as pulses of high resource availability are likely short-lived making strong responses to changing conditions a risky approach (e.g. DeWitt *et al.* 1998). We expect greater plasticity to variable resource conditions from southern populations coming from more productive, lower latitude environments than northern populations within the four experimental treatments. In addition, we hypothesize that southern populations will exhibit higher physiological rates and thus greater growth (e.g. biomass production, height growth) than northern populations under the ideal conditions of a controlled growth chamber experiment. We anticipate that enhanced access to a given resource will amplify these differences through strategic shifts in biomass allocation and changes in physiological rates (e.g. Oleksyn *et al.* 1998; Green 2005). By comparing the differences in an extensive suite of black spruce traits in a common garden, this study can help elucidate understanding of local adaptation vs. plasticity in the warming boreal forests of northwestern Canada.

## Methods

### Germination and planting

Black spruce seed was acquired from five locations across the latitudinal extent of the species in western Canada (Fig. 1). The 14-month-long experiment began in July 2014. After a period of cold stratification, non-viable seeds were removed by performing a float in 70 % ethanol, which also served to surface-sterilize the seeds. Seeds were plated by population in 60 × 15 mm petri dishes of 1 % agar and 0.2 g L^−1^ of liquid fertilizer (Miracle-Gro 24-8-16 Water Soluble All Purpose Plant Food, Miracle-Gro Lawn Products, Inc., Marysville, OH, USA), with ~20 seeds per plate. The plated seeds were maintained in growth chambers (Bigfoot models LTCB-19 and TPC-19; BioChambers Inc., Winnipeg, MB, Canada) at a 16/8 h dark/light cycle at 20 °C.

After 2 weeks, 60 germinants each from the Prince Albert, Fort McMurray and Inuvik populations were individually transplanted into 1-L Treepots (5 × 5 × 30 cm, Stuewe & Sons, Inc., Tangent, OR, USA) containing a mixture of 50 % potting soil (0.18-0.1-0.1 Miracle-Gro Moisture Control Potting Mix, Miracle-Gro Lawn Products, Inc., Marysville, OH, USA; equivalent to 465.77 g N m^−2^) and 50 % Turface (Turface Athletics MVP, Profile Products LLC, Buffalo Grove, IL, USA) with 3 mL of ectomycorrhizal inoculum (MYKE PRO LANDSCAPE, Premier Tech Ltd, Rivière-du-Loup, QC, Canada) surrounding the seed to aid in successful establishment of the seedlings (Lamhamedi and Bernier 1994). Poor germination of the Fort Simpson and Norman Wells populations led to only 24 and 19 germinated seeds being planted, respectively. These populations were excluded from the remainder of the experiment due to insufficient replication.

### Experimental design

Our experiment consisted of four treatments in a fully factorial design: elevated CO_2_ (EC), ambient CO_2_ (AC), high nutrient (nitrogen–phosphorus–potassium fertilizer; HN) and low nutrient (LN). Plants with assigned HN treatments were given 3 mL of slow release fertilizer mixed throughout the upper 15 cm of the soil column in the pot (equivalent to an additional 8.57 g N m^−2^ greater than the potting soil; Miracle-Gro Multi-Purpose Shake N Feed 10-10-10, Miracle-Gro Lawn Products, Inc., Marysville, OH, USA). The magnitude of fertilizer applied was based on the application instructions for the fertilizer; we were interested in increasing resource availability at a moderate rate rather than emulating natural conditions.

#### Growing conditions.

All plants were kept at ambient CO_2_ (~400 ppm) for the first 4 months of the experiment to allow seedlings to establish. This permitted us to then randomly assign surviving plants to eight blocks (using a random number generator) within two CO_2_ chambers—maximizing the number of living plants in both levels of CO_2_ treatments. All AC seedlings were grown in a growth chamber at ambient CO_2_ conditions (average 400 ppm), while EC plants were kept in a growth chamber where the CO_2_ concentration was maintained at an average of 750 ppm (the projected atmospheric CO_2_ concentrations for 2100 in models presented by the IPCC—www.ipcc-data.org/observ/ddc_co2) using Sentinel PPM controller (CPPM-4) and CO_2_ regulator (Sentinel Global Product Solutions, Inc., Santa Rosa, CA, USA). The CO_2_ was scrubbed using a potassium permanganate scrubber as described in Morison and Gifford (1984) to remove any potential organic contaminates such as ethylene that might influence growth. Although we recognize that it is not ideal to restrict all EC plants to one growth chamber while maintaining AC plants in one separate chamber, it is an accepted standard given the logistical constraints of elevating CO_2_ in an experimental setting with extensive replication (e.g. Bazzaz *et al.* 1990; Tjoelker *et al.* 1998; Way *et al.* 2010).

Following the seedling establishment period, the elevated CO_2_ treatment began. We simulated seasonal shifts over shorter than natural time periods, which have been proven successful for triggering bud set, chilling requirements, and growth initiation in boreal spruce seedling experiments from across latitudinal ranges (Johnsen and Seiler 1996; Mcleod 2001; Bigras and Bertrand 2006). During the growing season cycles the growth chambers were kept at a 16-h light cycle, with 22 °C/18 °C day/night temperatures. These conditions were maintained for 3 months before initiating a ‘winter’ cycle, during which temperatures and hours of light were gradually reduced over the course of 17 days to bring the chambers down to 4 °C and complete darkness. This 4 °C dark ‘winter’ cycle lasted for 3 weeks, at which point the temperature and light were gradually increased over 17 days back to the conditions of the growing period. We repeated this cycle after each 3-month growing season, for a total of three complete growing seasons over the course of the experiment. The growth chamber conditions chosen were not designed to emulate natural environmental conditions, rather they were to provide non-stressful conditions for plant growth.

During the growing period, trees were watered twice weekly until water was observed to drain out the bottom of the pots, and 1.5 mL of slow release fertilizer (equivalent to 60 g N m^−2^; Miracle-Gro Multi-Purpose Shake N Feed, 10-10-10) was added to all HN plants after each winter cycle to maintain the HN conditions. We added 0.5 mL of slow release fertilizer (equivalent to 20 g N m^−2^; Miracle-Gro Multi-Purpose Shake N Feed, 10-10-10) to the LN seedlings after the second winter because we began to notice signs of nutrient limitation.

#### Monthly measurements.

Each month of the growing period, we took measurements of seedling height and visible root length. Height was measured as the distance from the soil surface to the apical shoot tip. Root lengths were measured using by tracing visible roots in 5 × 15 cm windows cut into the front of each pot **[see Supporting Information—Fig. S1]**. The tracings were scanned and root lengths were measured using IJ_Rhizo (Pierret *et al.* 2013), an image analysis macro in ImageJ (version 1.50b).

#### Gas exchange measurements.

At the end of the 14-month experiment, we took a series of gas exchange measurements on four randomly selected seedlings from each surviving population and each CO_2_ treatment using a LI-6400 XT (LI-COR Environmental, Lincoln, NE, USA). We used the lighted conifer chamber when seedlings were large enough for a signal to be detected in this chamber. The seedlings were clamped into the chamber so as to fit as much of the plant from the apical meristem down, on fully expanded leaf tissue. For seedlings too small for the conifer chamber, a 2 × 3 chamber with a light source was used, placing the plant into the chamber using the same approach as above with all parameters maintained between the two chambers. Light response curves were created by measuring gas exchange (CO_2_ assimilation rate—µmol m^−2^ s^−1^) at increasing light levels (0, 20, 50, 100, 400, 600, 800, 1200, 2000 µmol m^−2^ s^−1^) with sufficient time for all readings to stabilize. Measurements were taken at CO_2_ concentrations of both 400 and 750 ppm to emulate the CO_2_ conditions experienced under the two experimental CO_2_ treatments. The gas exchange data were post-corrected for actual fresh leaf area. We fit modified Michaelis–Menten models to the PAR vs. CO_2_ assimilation rate data using the following equation:

$$A=\frac{A_{max}\;\times \;PAR}{\left(K_{m}+PAR\right)}$$ (1)

where *A* is the photosynthetic rate, *A*_max_ is the maximum photosynthetic rate, PAR is the intensity of photosynthetically active radiation (µmol m^−2^ s^−1^) and *K*_m_ is the Michaelis–Menten constant. These were implemented with the nls() function in R (R Core Team 2014), which allowed us to solve for the dark respiration rate (*R*_d_—µmol m^−2^ s^−1^; *A* where PAR is equal to 0), maximum photosynthetic rate (*A*_max_; µmol m^−2^ s^−1^) and leaf-level light compensation point (LCP—µmol m^−2^ s^−1^; PAR where *A* is equal to 0) under both 400 and 750 ppm CO_2_ concentration (indicated by subscripts 400 and 750, respectively).

#### Post-harvest measurements.

After all gas exchange measurements were made, the seedlings were harvested. We measured dry root biomass (Biomass_root_), dry leaf biomass (Biomass_leaf_), dry stem biomass (Biomass_stem_), dry total biomass (Biomass_total_), total root length (TRL; using WinRhizo version 2012b, Regent Instruments Inc., Quebec, QC, Canada) and total leaf area (TLA; using WinSeedle version 2004a, Regent Instruments Inc., Quebec, QC, Canada). Total root length and TLA were measured on fresh tissue. We also investigated allocation patterns in the seedlings by calculating specific leaf area (SLA; fresh leaf area divided by dry leaf mass), specific root length (SRL; fresh root length divided by dry root mass), root/shoot ratio (R:S), root/mass ratio (RMR), leaf/mass ratio (LMR) and stem/mass ratio (SMR). These abbreviations are defined in **Supporting Information—Table S1**, for reference throughout the article.

### Statistical analyses

All statistical analyses were performed in R version 3.1.2 (R Core Team 2014). Throughout the analyses, assumptions of normality, linearity and homoscedasticity were assessed visually using histograms, residual vs. fits plots and q-q plots, respectively. In our experiment, extensive mortality of seedlings in the HN treatment prevented its inclusion into all analyses of *a priori* hypotheses regarding growth, allocation and physiological traits. However, the mortality trends temporally and among populations led us to believe that these patterns reflected potential plastic responses. This hypothesis and analysis are described later in this section.

Root length and seedling height from treatments over time were compared by fitting linear mixed effect models in the package lme4 (Bates *et al.* 2015), with population, CO_2_ treatment and time as fixed effects, height or root length as the response and individual nested in block as the random effect term. Plant height and root length were log-transformed in order to meet assumptions of normality. We explored interaction effects of population, CO_2_ treatment and time in both the height and root length models.

Gas exchange and post-harvest measurements were compared using linear mixed effect models in the package lme4 (Bates *et al.* 2015), with CO_2_ treatment and population as fixed effects and block as a random effect. Log-transformations were applied to LCP_750_, LCP_400_, Biomass_root_, Biomass_leaf_, Biomass_stem_, Biomass_above_, Biomass_total_, TRL, TLA, SLA, R:S and SMR in order to meet assumptions of normality. Tukey HSD tests were used to test for significant differences between populations and CO_2_ treatments. For all above analyses, the package ‘lmerTest’ (Kuznetsova *et al.* 2016) was used to calculate Satterthwaite approximations of the denominator degrees of freedom in order to perform ANOVA, and *post hoc* pairwise comparisons were performed where necessary for interpretation using the package ‘lsmeans’ (Lenth 2016).

We conducted a survival analysis of the seedlings in the experiment, including the HN treatment. This analysis was prompted when we unexpectedly observed some seedlings with high resource treatments exhibiting two growth flushes in a single growing season, and consequently not completely hardening prior to the onset of winter. Survival analysis was performed using Kaplan–Meier survival estimates in the ‘survival’ package in R (Therneau 2015). To determine differences in cumulative survival of each treatment, we implemented a log-rank test using the function survdiff().

## Results

### Monthly measurements

**Figure 2.**
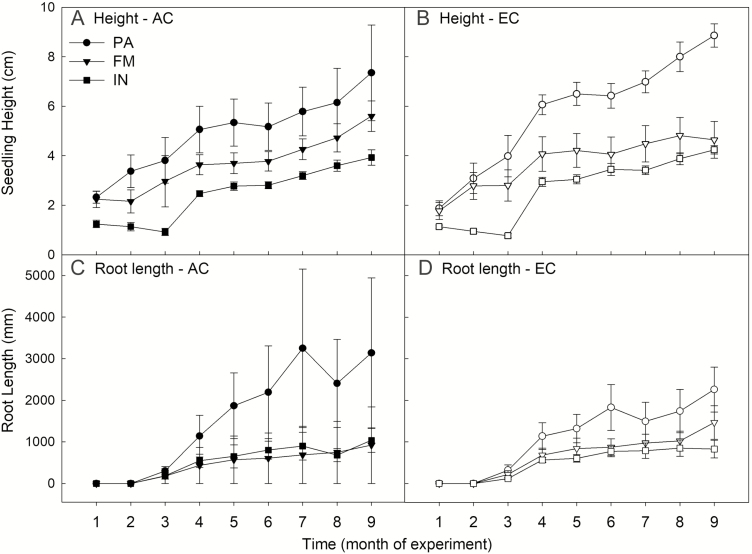
Mean seedling height (A, B) and root length (C, D) over the course of the experiment. Each time point corresponds to our monthly measurements throughout three 3-month growing seasons (season 1: 1–3, season 2: 4–6, season 3: 7–9). Populations shown are Prince Albert SK (PA), Fort McMurray AB (FM) and Inuvik NT (IN). Ambient CO_2_ treatment is represented by filled symbols (AC), while elevated CO_2_ is shown as unfilled symbols (EC). Error bars represent SE. There were significant differences between the height of the three populations throughout the experiment, and how population heights change over time (results shown in Table 1), while CO_2_ treatment did not significantly affect height growth. Neither population nor treatment was a significant term in the model for root length throughout the experiment.

Over the course of the experiment, we found a significant interaction between population and time in our model of seedling height, driven by the fact that southern populations grew at a faster rate than northern populations (Fig. 2; Table 1). Although we found a significant interaction between CO_2_ treatment and time in our model (Table 1), *post hoc* testing of EC vs. AC height within each time period did not identify significant contrasts **[see Supporting Information—Table S2]**. The patterns we observed reflect the latitudinal gradient captured by the study; the seedlings from Inuvik were consistently the shortest, while tallest seedlings were found in the Prince Albert population (Fig. 2A and B; Table 1). In contrast, we found no effect of population or treatment on root length (from our monthly root windows) throughout the experiment (Table 2)—the only significant term in our model was time (*F*_1, 225_ = 203.34, *P* < 0.0001), which simply demonstrates root growth over time (Fig. 2C and D).

**Table 1.** ANOVA table for the linear mixed effects model of black spruce seedling height from three populations (Prince Albert SK, Fort McMurray AB and Inuvik NT) over the course of the experiment. Individual nested in block was included as a random effect. Denominator degrees of freedom were calculated using a Satterthwaite approximation.

	Sum of squares	Mean squares	Numerator d.f.	Denominator d.f.	*F*-value	*P*-value
Population	4.097	2.049	2	38.330	23.230	<0.0001
CO_2_	0.051	0.051	1	37.560	0.577	n.s.
Time	79.513	9.939	8	328.720	112.713	<0.0001
Population * CO_2_	0.031	0.016	2	38.740	0.176	n.s.
Population * Time	9.996	0.625	16	328.740	7.085	<0.0001
CO_2_ * Time	1.551	0.194	8	328.720	2.198	0.027
Population * CO_2_ * Time	1.564	0.0977	16	328.730	1.108	n.s.

**Table 2.** ANOVA table for the linear mixed effects model of black spruce seedling root length measurements from three populations (Prince Albert SK, Fort McMurray AB and Inuvik NT) over the course of the experiment. Individual nested in block was included as a random effect. Denominator degrees of freedom were calculated using a Satterthwaite approximation.

	Sum of squares	Mean squares	Numerator d.f.	Denominator d.f.	*F*-value	*P*-value
Population	0.461	0.231	2	40.279	1.583	n.s.
CO_2_	0.093	0.0934	1	40.278	0.641	n.s.
Time	86.287	14.3811	6	192.225	98.749	<0.0001
Population * CO_2_	0.101	0.051	2	40.279	0.348	n.s.
Population * Time	1.628	0.136	12	192.204	0.931	n.s.
CO_2_ * Time	0.729	0.122	6	192.225	0.834	n.s.
Population * CO_2_ * Time	0.974	0.081	12	192.204	0.557	n.s.

### Gas exchange measurements

**Figure 3.**
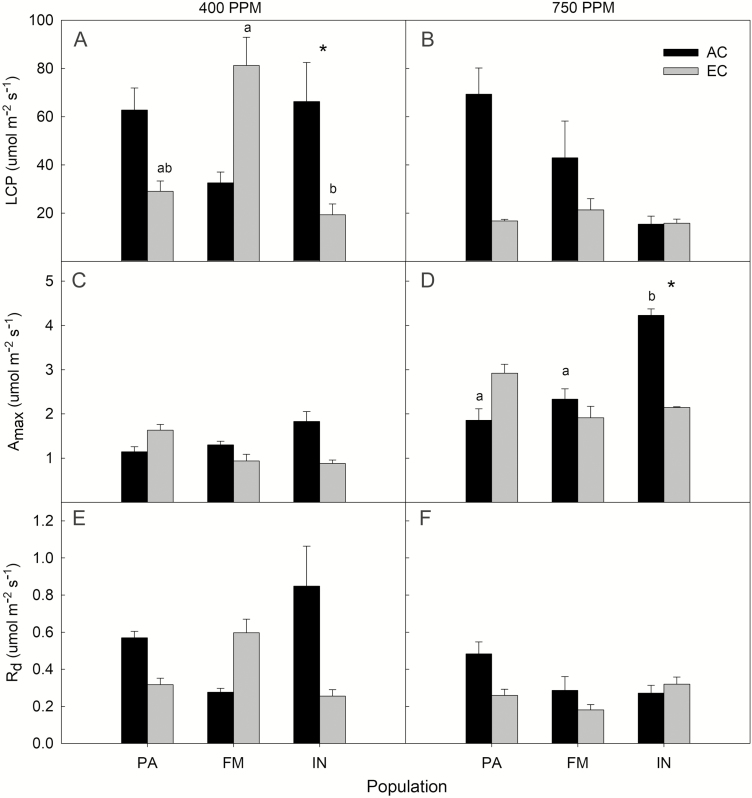
Light compensation point (LCP), maximum photosynthetic rate (*A*_max_) and dark respiration (*R*_d_) for each of the populations (IN—Inuvik NT; FM—Fort McMurray AB; PA—Prince Albert SK) and CO_2_ treatments (AC—ambient CO_2_; EC—elevated CO_2_). The CO_2_ concentrations under which the gas exchange measurements were made are indicated by the titles above each panel column (400 or 750 ppm). Error bars indicate SE around the means. Significant differences between AC/EC treatments with a population are denoted by (*). Within each CO_2_ treatment, significant differences between populations are indicated by letter codes.

Physiological rates were surprisingly invariable in our study; we found no significant effect of either population or CO_2_ treatment on dark respiration rate (*R*_d_, at 400 or 750 ppm), LCP (at 750 ppm) or maximum photosynthetic rate (*A*_max_; at 400 ppm). However, we found a significant interaction between population and CO_2_ treatment on LCP at 400 ppm (*F*_2, 18_ = 3.67, *P* = 0.046). Under these conditions, the EC treatment of Fort McMurray had a significantly greater LCP than Inuvik seedlings, and within the Inuvik population, LCP of AC seedlings was significantly higher than EC seedlings (Fig. 3). Measurements of *A*_max_ at 750 ppm had a significant interaction between population and CO_2_ treatment (*F*_2, 18_ = 7.44, *P* = 0.0044), and a significant main effect of population (*F*_2, 18_ = 3.71, *P* = 0.045). Within the AC treatment, Inuvik seedlings had a significantly greater *A*_max_ than the other two populations, and within the Inuvik seedlings *A*_max_ was greater in AC vs. EC treatments (Fig. 3).

### Post-harvest measurements

**Figure 4.**
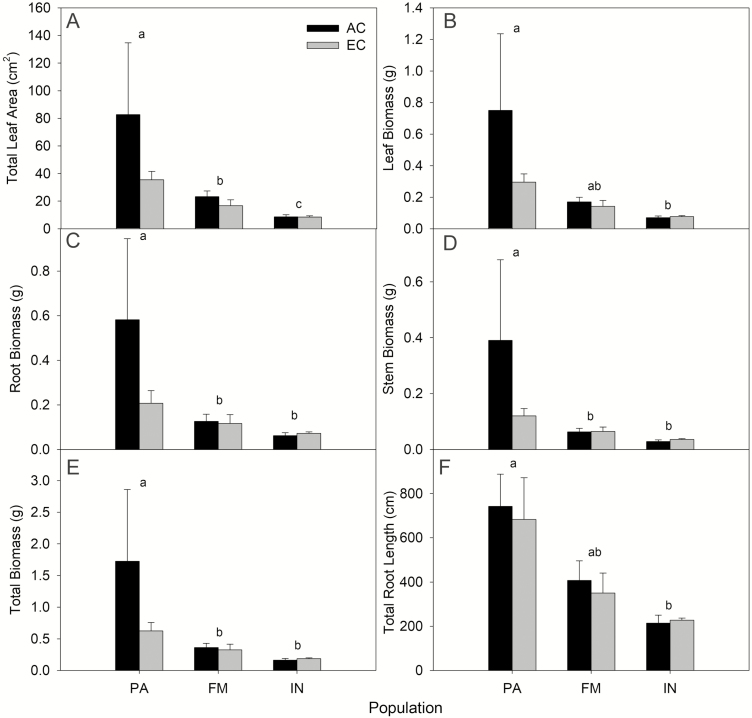
Post-harvest morphological trait measurements that exhibit significant differences. Populations shown are Prince Albert SK (PA), Fort McMurray AB (FM) and Inuvik NT (IN) and CO_2_ treatments are ambient CO_2_ (AC) and elevated CO_2_ (EC). Error bars indicate SE around the means. Treatments showing significant differences are indicated by different letter codes.

There were many population-level differences in the physical traits measured post-harvest. Total leaf area was significantly different among all three populations, with increased leaf area in seedlings moving from northern to southern populations (Fig. 4; *F*_2, 29_ = 16.343, *P* < 0.0001). Leaf biomass and TRL also decreased with latitude; however, the Fort McMurray population was not significantly different from either the Prince Albert or Inuvik populations (Fig. 4; Biomass_leaf_—*F*_2, 29_ = 15.110, *P* < 0.0001; TRL—*F*_2, 29_ = 8.237, *P* = 0.0015). Root biomass, stem biomass and total biomass were all significantly greater in Prince Albert seedlings than Fort McMurray and Inuvik (Fig. 4; Biomass_root_—*F*_2, 29_ = 9.241, *P* < 0.001; Biomass_stem_—*F*_2, 29_ = 12.961, *P* < 0.0001; Biomass_total_—*F*_2, 29_ = 13.745, *P* < 0.0001). The CO_2_ treatment had no significant effect on the outcomes of the aforementioned traits.

However, despite the population-level differences that followed a latitudinal trend, allocation traits were not consistent across latitude. There was a significant difference in SLA among populations (*F*_2, 25_ = 4.006, *P* = 0.031) and CO_2_ treatment (*F*_1, 25_ = 4.480, *P* = 0.045). Specific leaf area was higher in Fort McMurray seedlings than Inuvik, and seedlings under the AC treatment had greater SLA than EC **[see Supporting Information—Fig. S2]**. We found no treatment effect on SRL, R:S, RMR, LMR and SMR **[see Supporting Information—Fig. S2]**.

### Survival analysis

**Figure 5.**
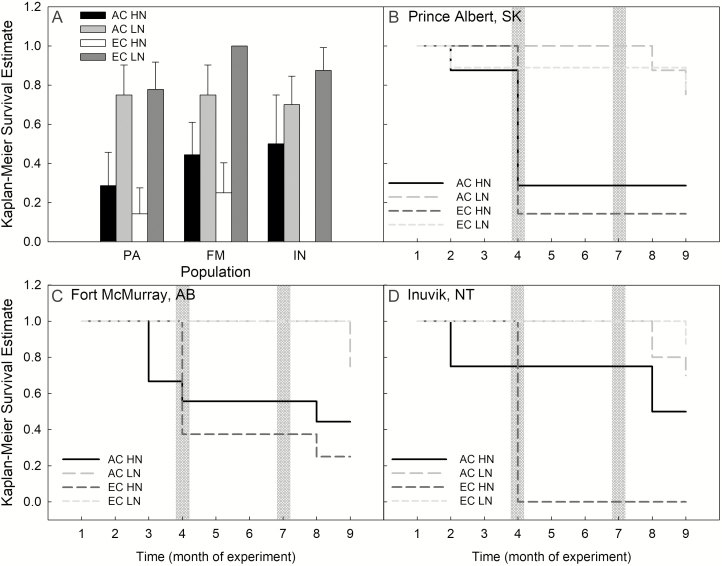
(A) Mean cumulative Kaplan–Meier survival estimate of seedlings under each treatment in the experiment. (B–D) Survival curves showing Kaplan–Meier survival estimates throughout the experiment for each population. Populations shown are Prince Albert SK (PA), Fort McMurray AB (FM) and Inuvik NT (IN), CO_2_ treatments are ambient CO_2_ (AC) and elevated CO_2_ (EC), and nutrient treatments are low nutrient (LN) and high nutrient (HN). Error bars indicate SE around the means. Grey vertical bars on panels B–D indicate the first month of measurements post-winter. Each time point corresponds to our monthly measurements throughout three 3-month growing seasons (season 1: 1–3, season 2: 4–6, season 3: 7–9).

We found that there was a significant difference in survival between treatments—HN seedlings had consistently lower survival than their LN counterparts (Fig. 5; Χ^2^ = 38.7, d.f. = 11, *P* ≤ 0.0001).

## Discussion

In this study, we found strong evidence for genetic differentiation among the populations studied through the very distinct latitudinal patterns in growth-related traits (e.g. height, biomass, root length, etc.). Seedlings from Prince Albert (the southernmost provenance) were always largest in height, biomass and root length measurements, with seedling size exhibiting a decreasing trend moving northward (Fig. 4). This pattern was not maintained among allocation and physiological traits—there were few significant differences across measurements, and no consistent latitudinal trend was discernable across this suite of measurements (Fig. 3; **see Supporting Information—Fig. S2**). There was not strong evidence for plasticity in the responses to elevated CO_2_—the only traits which exhibited significant differences between the AC and EC treatment were LCP (at 400 ppm), maximum photosynthetic rate (*A*_max_; at 750 ppm) and SLA (Fig. 2; **see Supporting Information—Fig. S2**). However, in the survival analysis, we found that mortality was greater in the HN treatments. Observations throughout the experiment lead us to hypothesize that this result is indicative of some plastic responses to high resource availability in some phenological traits, as we explain below.

### Evidence for genetic differentiation between populations

#### Growth traits and the fast–slow spectrum.

Consistent with our hypotheses, we found that—under the same conditions—the southernmost population (Prince Albert) had greater height and biomass production, leaf area and root length than the more northerly populations (Fig. 4). At the southernmost extent of black spruce, trees are competing for resources with many faster-growing tree species than at northern sites (e.g. Bell *et al.* 2000). The parent trees of these seedlings would also have been accustomed to longer, warmer growing seasons than northern populations, making them likely candidates to exhibit a fast trait strategy. *In situ*, we would expect southern populations to be able to take advantage of increasing resource availability in a changing climate. At the species’ northernmost extent, growth is constrained to a very short frost-free growing season. For northern populations, a conservative slow trait strategy in which individuals produce new biomass early, harden new growth and set bud before severe weather strikes should facilitate persistence under the extreme conditions at northernmost extents of forest (e.g. DeWitt *et al.* 1998). Although we found contrasting results in root length between the monthly (no significant population differences; Fig. 2) and end of experiment (significant population differences; Fig. 4) analyses, this is likely a result of the lack of differentiation between populations during the first few months of the experiment. During this time, there were few roots visible in the root windows—differences in root length between populations by month became more evident at the end of the experiment, and it is reflected in the TRL measurements post-harvest.

#### Physiological and allocation traits.

Population-level differences were not apparent in our measures of allocation and physiological traits. We found that the Inuvik seedlings had a significantly higher maximum photosynthetic rate (*A*_max_; measured at 750 ppm) than the two southern populations (Fig. 3D), which is consistent with studies of black spruce gas exchange across large latitudinal extents (Bigras and Bertrand 2006). We found that the magnitude of *A*_max_ observed in our experiment (0.88–4.23 µmol m^−2^ s^−1^) was overlapping but lower than other studies of black spruce, which ranged from 2.5 to 25 µmol m^−2^ s^−1^ (Tjoelker *et al.* 1998; Bigras and Bertrand 2006; Way and Sage 2008). However, the seed stock was all sourced from provenances in Ontario, Quebec and Minnesota—all of which are considerably southeast of our study region. Differences between the large-scale climate patterns and environmental pressures (e.g. competition) between the western and eastern boreal forests may drive different strategies that could have led to this contrasting result.

All other physiological measurements did not exhibit a latitudinal trend that would further support the clear genetic differentiation observed in the growth traits (Fig. 3). Allocation trait measurements were similarly inconclusive. Although there was a significant difference in SLA among some of the populations, there was no apparent latitudinal trend. All other comparisons of allocation traits found no differences or consistent patterns among populations **[see Supporting Information—Fig. S2]**. In contrast to our finding that R:S (and other allometric relationships) was not significantly different among populations and treatments, Johnsen and Seiler (1996) observed greater R:S in northern provenances of black spruce. This could be as a result of the longer (20 h) photoperiod implemented by Johnsen and Seiler (1996) that allowed for seedlings to develop more pronounced differences in their allocation patterns.

### Plasticity of traits in response to resource treatments

Similar to our findings, many studies on black spruce support our result that black spruce is not very plastic in many traits including phenology, biomass, height, gas exchange and wood anatomy in response to resource availability (Morgenstern and Mullin 1990; Parker *et al.* 1994; Johnsen and Seiler 1996; Johnsen *et al.* 1996; Beaulieu *et al.* 2004; Bigras and Bertrand 2006; Balducci *et al.* 2015). Experiments with similar CO_2_ treatments to our study found that black spruce responded to the elevated CO_2_ conditions through increases in height growth, shoot mass and non-structural carbohydrates as well as decreases in stomatal conductance, leaf nitrogen content and SLA (Tjoelker *et al.* 1998; Bigras and Bertrand 2006). However, it should be noted that these experiments did not indicate that the CO_2_ used to elevate the experimental CO_2_ conditions had been scrubbed of ethylene—a plant growth hormone that can result in effects such as increased shoot elongation and diameter (Abeles 1971), and it is a known contaminant in CO_2_ cylinders (Morison and Gifford 1984). There is also evidence to support no effect of CO_2_ on physiological and morphological traits in previous studies (Johnsen and Seiler 1996). The different responses observed could be due to differences between eastern provenances and those used in our study, or acclimation to increased CO_2_ conditions over the course of the experiment as observed by Johnsen and Seiler (1996).

Although we attempted to characterize plastic vs. fixed traits under different nutrient treatments, poor survival of the HN treatment after our first simulated ‘winter’ prevented further inclusion of this treatment. However, we found that our HN treatment had significantly lower survival than seedlings under LN treatments (Fig. 5). We interpret the poor survival of our HN seedlings not as nutrient toxicity (N toxicity in black spruce seedlings occurs at 2000 mg N L^−1^ soil (Salifu and Timmer 2003) in comparison to our 905 mg N L^−1^), but instead as support for some degree of plasticity in the strategies of seedlings. During the experiment, the greatest deaths occurred during the first ‘winter’ cycle (Fig. 5B–D). We observed that the majority of the HN seedlings continued to grow new shoots throughout the 3-month ‘summer’ cycle and had not hardened new needles or set bud prior to the transition to the first ‘winter’. Over this time, we found that the HN treatment promoted height growth in comparison to the LN treatment **[see Supporting Information—Fig. S3]**. However, HN seedlings suffered severe damage over winter, and ultimately led to the widespread mortality recorded in the HN plants. This trend was most apparent in the HN × EC treatment, and in the two southerly populations (Fig. 5A). Several studies have shown that nutrient additions have significant impacts on tree phenology (Bigras *et al.* 1996), especially in seedlings undergoing elevated CO_2_ treatments (Murray *et al.* 1994; Sigurdsson 2001). In particular, high levels of nitrogen fertilization are known to reduce cold hardiness over winter and delay bud set (van den Driessche 1991). Thus, we believe the mortality we observed to be part of the plastic response of black spruce in growth and bud set strategies.

Depending on future climate patterns, this plastic response could benefit black spruce by allowing it to take advantage of lengthening growing season. Alternatively, if there is greater variability in weather patterns (particularly in autumn), delayed bud set may lead to increased risk of frost damage and reduced productivity. Unfortunately, our experiment was not designed to examine these effects in detail. However, this hypothesis has important implications for the future of the boreal forest—particularly those on permafrost where thaw is expected to increase nutrient availability (e.g. Keuper *et al.* 2012)—and should be explored in future experiments.

## Conclusions

In this study, we found evidence of local adaptation among the populations, as there were significant trends in growth rates and many of the morphological traits measured in this experiment. These differences were not reflected in the allocation traits or among most of the physiological traits. Our unexpected survival data generated a novel hypothesis that under enhanced resource conditions, black spruce seedlings may delay bud set, putting them at greater risk of winter damage. Because we cannot confirm or deny the extent of plasticity through the results of this experiment, reciprocal transplant studies are required to be able to make predictions about the future of the boreal forest as climate change continues (e.g. Sultan 2000). In addition, studies on local adaptation and trait plasticity of mature trees are also important to filling gaps in our understanding of species resilience, as there is considerable evidence of trees showing contrasting responses to warming and resource availability between ontogenetic stages (Chung *et al.* 2013; Camarero *et al.* 2015). However, our study has examined an extensive suite of morphological, allocation and physiological traits across an area of the boreal forest where black spruce traits have remained largely understudied. Trait plasticity has the potential to provide a degree of resilience under changing conditions. Thus, filling these gaps and determining the potential for populations of this dominant boreal tree species to demonstrate plastic responses to variable resource conditions are crucial to predicting the resilience of black spruce forests to ongoing climate change in the boreal region. The results of this experiment provide an important first look at populations in this area and how they may respond to the extreme warming that is predicted for northwestern Canada.

## Sources of Funding

Funding for this research was provided by the Natural Sciences and Engineering Research Council, the Changing Cold Regions Network, the Canada Foundation for Innovation, the Government of the Northwest Territories and the Ontario Ministry of Research and Innovation Early Researcher Award program. A.E.S. was supported by Ontario Graduate Scholarships and G.G.M. by a Banting Fellowship.

## Contributions by the Authors

J.L.B. and G.G.M. developed the idea for the experiment. All authors developed methodology. G.G.M. and A.E.S. performed the experiment. A.E.S. analysed the data and wrote the manuscript; all other authors provided input on analytical methods and editorial support.

## Conflict of Interest

None declared.

## Supporting Information

The following additional information is available in the online version of this article—

**Table S1.** List of traits studied, and acronyms used for these traits.

**Table S2.** Results of pairwise contrasts of height growth model between AC and EC treatments over the course of the experiment.

**Figure S1.** Schematic of the ‘windows’ created in seedling pots to monitor root growth measurement throughout the experiment. The window was created by inserting a transparency sheet into the pot.

**Figure S2.** Post-harvest allocation trait measurements. Populations shown are Prince Albert SK (PA), Fort McMurray AB (FM) and Inuvik NT (IN) and CO_2_ treatments are ambient CO_2_ (AC) and elevated CO_2_ (EC). Error bars indicate SE around the means. Significant differences between populations are indicated by letters. Significant differences between CO_2_ treatments are denoted by an asterisk (*) in the upper right corner of the plot.

**Figure S3.** Mean seedling height over the first 3-month-long growing season. Populations shown are Prince Albert SK (PA), Fort McMurray AB (FM) and Inuvik NT (IN). Ambient CO_2_ treatment is represented by filled symbols (AC), while elevated CO_2_ is shown as unfilled symbols (EC). Error bars represent SE.

## Supplementary Material

Supplementary Material

## References

[CIT0001] Abeles F 1971 The role of ethylene in plant growth and development. Frederick, MD: Department of the Army.

[CIT0002] Aitken SN, Yeaman S, Holliday JA, Wang T, Curtis-McLane S 2008 Adaptation, migration or extirpation: climate change outcomes for tree populations. Evolutionary Applications 1:95–111. 25567494 10.1111/j.1752-4571.2007.00013.x PMC3352395

[CIT0003] Allen CD, Breshears DD, McDowell NG 2015 On underestimation of global vulnerability to tree mortality and forest die-off from hotter drought in the Anthropocene. Ecosphere 6:129.

[CIT0004] Balducci L, Deslauriers A, Giovannelli A, Beaulieu M, Delzon S, Rossi S, Rathgeber CB 2015 How do drought and warming influence survival and wood traits of *Picea mariana* saplings?Journal of Experimental Botany 66:377–389. 25371502 10.1093/jxb/eru431 PMC4265170

[CIT0005] Bates D, Maechler M, Bolker B, Walker S 2015 Fitting linear mixed-effects models using lme4. Journal of Statistical Software 67:1–48.

[CIT0006] Bazzaz FA, Coleman J, Morse SR 1990 Growth responses of seven major co-occurring tree species of the northeastern United States to elevated CO_2_. Canadian Journal of Forest Research 20:1479–1484.

[CIT0007] Beaulieu J, Perron M, Bousquet J 2004 Multivariate patterns of adaptive genetic variation and seed source transfer in *Picea mariana*. Canadian Journal of Forest Research 34:531–545.

[CIT0090] Bell FW, Ter-Mikaelian MT, Wagner RG 2000 Relative competitiveness of nine early-successional boreal forest species associated with planted jack pine and black spruce seedlings. Canadian Journal of Forest Research 30: 790–800.

[CIT0008] Benito Garzón M, Alía R, Robson TM, Zavala MA 2011 Intra-specific variability and plasticity influence potential tree species distributions under climate change. Global Ecology and Biogeography 20:766–778.

[CIT0009] Bigras FJ, Bertrand A 2006 Responses of *Picea mariana* to elevated CO_2_ concentration during growth, cold hardening and dehardening: phenology, cold tolerance, photosynthesis and growth. Tree Physiology 26:875–888. 16585033 10.1093/treephys/26.7.875

[CIT0010] Bigras FJ, Gonzalez A, Aoust ALD, Hebert C 1996 Frost hardiness, bud phenology and growth of containerized *Picea mariana* seedlings grown at three nitrogen levels and three temperature regimes. New Forests 12:243–259.

[CIT0011] Bonan GB, Shugart HH 1989 Environmental factors and ecological processes in boreal forests. Annual Review of Ecology and Systematics 20:1–28.

[CIT0012] Camarero JJ, Gazol A, Galván JD, Sangüesa-Barreda G, Gutiérrez E 2015 Disparate effects of global-change drivers on mountain conifer forests: warming-induced growth enhancement in young trees vs. CO_2_ fertilization in old trees from wet sites. Global Change Biology 21:738–749. 25362899 10.1111/gcb.12787

[CIT0013] Chapin FS 3rd, Sturm M, Serreze MC, McFadden JP, Key JR, Lloyd AH, McGuire AD, Rupp TS, Lynch AH, Schimel JP, Beringer J, Chapman WL, Epstein HE, Euskirchen ES, Hinzman LD, Jia G, Ping CL, Tape KD, Thompson CD, Walker DA, Welker JM 2005 Role of land-surface changes in arctic summer warming. Science 310:657–660. 16179434 10.1126/science.1117368

[CIT0014] Chung H, Muraoka H, Nakamura M, Han S, Muller O, Son Y 2013 Experimental warming studies on tree species and forest ecosystems: a literature review. Journal of Plant Research 126:447–460. 23689840 10.1007/s10265-013-0565-3

[CIT0015] Dewitt TJ, Sih A, Wilson DS 1998 Costs and limits of phenotypic plasticity. Trends in Ecology & Evolution 13:77–81. 21238209 10.1016/s0169-5347(97)01274-3

[CIT0016] van den Driessche R 1991 Effects of nutrients on stock performance in the forest In: van den Driessche R, ed. Mineral nutrition of conifer seedlings. Boca Raton, FL: CRC Press, 229–260.

[CIT0017] Flato GM, Boer GJ 2001 Warming asymmetry in climate change simulations. Geophysical Research Letters 28:195–198.

[CIT0018] Green DS 2005 Adaptive strategies in seedlings of three co-occurring, ecologically distinct northern coniferous tree species across an elevational gradient. Canadian Journal of Forest Research 35:910–917.

[CIT0019] Hartmann DL, Klein Tank AMG, Rusticucci M, Alexander LV, Brönnimann S, Charabi Y, Dentener FJ, Dlugokencky EJ, Easterling DR, Kaplan A, Soden BJ, Thorne PW, Wild M, Zhai PM. 2013 Observations: Atmosphere and Surface. In: Stocker TF, Qin D, Plattner G-K, Tignor M, Allen SK, Boschung J, Nauels A, Xia Y, Bex V, Midgley PM eds. Climate Change 2013: The Physical Science Basis. Contribution of Working Group I to the Fifth Assessment Report of the Intergovernmental Panel on Climate Change. Cambridge, United Kingdom and New York, NY, USA: Cambridge University Press.

[CIT0020] Johnsen KH, Seiler JR 1996 Growth, shoot phenology and physiology of diverse seed sources of black spruce: I. Seedling responses to varied atmospheric CO_2_ concentrations and photoperiods. Tree Physiology 16:367–373. 14871738 10.1093/treephys/16.3.367

[CIT0021] Johnsen KH, Seiler JR, Major JE 1996 Growth, shoot phenology and physiology of diverse seed sources of black spruce: II. 23-year-old field trees. Tree Physiology 16:375–380. 14871739 10.1093/treephys/16.3.375

[CIT0022] Jump AS, Peñuelas J 2005 Running to stand still: adaptation and the response of plants to rapid climate change. Ecology Letters 8:1010–1020. 10.1111/j.1461-0248.2005.00796.x 34517682

[CIT0023] Keeling CD, Chin JFS, Whorf TP 1996 Increased activity of northern vegetation inferred from atmospheric CO_2_ measurements. Nature 382:146–149.

[CIT0024] Keuper F, van Bodegom PM, Dorrepaal E, Weedon JT, van Hal J, van Logtestijn RSP 2012 A frozen feast: thawing permafrost increases plant-available nitrogen in subarctic peatlands. Global Change Biology 18:1998–2007.

[CIT0025] Kuznetsova A, Brockhoff PB, Christensen RHB 2016 *lmerTest: tests in linear mixed effects models. R package version 2.0-33* https://CRAN.R-project.org/package=lmerTest. (10 December 2016)

[CIT0026] Lamhamedi M, Bernier P 1994 Ecophysiology and field performance of black spruce (*Picea mariana*): a review. Annals of Forest Science 51:529–551.

[CIT0027] Lenth RV 2016 Least-squares means: the R package lsmeans. Journal of Statistical Software 69:1–33.

[CIT0028] Mcleod TK 2001 The ecology of *Picea glauca* (Moench) Voss at its range limits in northwest Canada, University of British Columbia.

[CIT0029] Morgenstern EK, Mullin TJ 1990 Growth and survival of black spruce in the range-wide provenance study. Canadian Journal of Forest Research 20:130–143.

[CIT0030] Morison JI, Gifford RM 1984 Ethylene contamination of CO_2_ cylinders: effects on plant growth in CO_2_ enrichment studies. Plant Physiology 75:275–277. 16663594 10.1104/pp.75.1.275 PMC1066883

[CIT0031] Murray MB, Smith RI, Leith ID, Fowler D, Lee HS, Friend AD, Jarvis PG 1994 Effects of elevated CO_2_, nutrition and climatic warming on bud phenology in Sitka Spruce (*Picea sitchensis*) and their impact on the risk of frost damage. Tree Physiology 14:691–706. 14967641 10.1093/treephys/14.7-8-9.691

[CIT0032] Oleksyn J, Modrzynski J, Tjoelker MG, Zytkowiak R, Reich PB, Karolewski P 1998 Growth and physiology of *Picea abies* populations from elevational transects: common garden evidence for altitudinal ecotypes and cold adaptation. Functional Ecology 12:573–590.

[CIT0033] Parker WH, Niejenhuis AV, Charrette P 1994 Adaptive variation in *Picea mariana* from northwestern Ontario determined by short-term common environment tests. Canadian Journal of Forest Research 24:1653–1661.

[CIT0034] Pierret A, Gonkhamdee S, Jourdan C, Maeght JL 2013 IJ_Rhizo: an open-source software to measure scanned images of root samples. Plant and Soil 373:531–539.

[CIT0091] R Core Team. 2014. R: A language and environment for statistical computing. R Foundation for Statistical Computing, Vienna, Austria. https://www.R-project.org/ (23 November 2014).

[CIT0035] Reich PB 2014 The world-wide “fast-slow” plant economics spectrum: a traits manifesto. Journal of Ecology 102:275–301.

[CIT0036] Salifu KF, Timmer VR 2003 Optimizing nitrogen loading of *Picea mariana* seedlings during nursery culture. Canadian Journal of Forest Research 33:1287–1294.

[CIT0037] Savolainen O, Bokma F, García-Gil R, Komulainen P, Repo T 2004 Genetic variation in cessation of growth and frost hardiness and consequences for adaptation of *Pinus sylvestris* to climatic changes. Forest Ecology and Management 197:79–89.

[CIT0038] Scinocca JF, McFarlane NA, Lazare M, Li J, Plummer D 2008 Technical note: the CCCma third generation AGCM and its extension into the middle atmosphere. Atmospheric Chemistry and Physics 8:7055–7074.

[CIT0039] Sigurdsson BD 2001 Elevated [CO_2_] and nutrient status modified leaf phenology and growth rhythm of young *Populus trichocarpa* trees in a 3-year field study. Trees - Structure and Function 15:403–413.

[CIT0040] Sultan SE 1995 Phenotypic plasticity and plant adaptation. Acta Botanica Neerlandica 44:363–383.

[CIT0041] Sultan SE 2000 Phenotypic plasticity for plant development, function and life history. Trends in Plant Science 5:537–542. 11120476 10.1016/s1360-1385(00)01797-0

[CIT0042] Therneau T 2015 *A package for survival analysis in S. version 2.38* https://CRAN.R-project.org/package=survival. (12 January 2016)

[CIT0043] Tjoelker MG, Oleksyn J, Reich PB 1998 Seedlings of five boreal tree species differ in acclimation of net photosynthesis to elevated CO_2_ and temperature. Tree Physiology 18:715–726. 12651406 10.1093/treephys/18.11.715

[CIT0044] Vitasse Y, Hoch G, Randin CF, Lenz A, Kollas C, Scheepens JF, Körner C 2013 Elevational adaptation and plasticity in seedling phenology of temperate deciduous tree species. Oecologia 171:663–678. 23306445 10.1007/s00442-012-2580-9

[CIT0045] Walther GR, Post E, Convey P, Menzel A, Parmesan C, Beebee TJ, Fromentin JM, Hoegh-Guldberg O, Bairlein F 2002 Ecological responses to recent climate change. Nature 416:389–395. 11919621 10.1038/416389a

[CIT0046] Way DA, Ladeau SL, McCarthy HR, Clark JS, Oren R, Finzi AC, Jackson RB 2010 Greater seed production in elevated CO_2_ is not accompanied by reduced seed quality in *Pinus taeda* L. Global Change Biology 16:1046–1056.

[CIT0047] Way DA, Sage RF 2008 Elevated growth temperatures reduce the carbon gain of black spruce [*Picea mariana* (Mill.) B.S.P.]. Global Change Biology 14:624–636.

