# Supplementary material for: Assessing local adaptation vs. plasticity under different resource conditions in seedlings of a dominant boreal tree species
Source: AoB Plants. 2018 Jan 19;10(1):ply004. doi: 10.1093/aobpla/ply004 (PMC5811874; doi:10.1093/aobpla/ply004)
Supplement: Supplementary Material [file ply004_suppl_supplementary-material.docx]

**Supporting Information**

**Assessing local adaptation vs. plasticity under different resource conditions in seedlings of a dominant boreal tree species**

**Table S1.** List of traits studied, and acronyms used for these traits.

| Trait type | Acronym | Trait description |
| --- | --- | --- |
| Growth | TLA | Total Leaf Area |
|  | Biomass_leaf_ | Total Leaf Biomass |
|  | Biomass_root_ | Total Root Biomass |
|  | Biomass_stem_ | Total Stem Biomass |
|  | Biomass_total_ | Total Biomass |
|  | TRL | Total Root Length |
|  | n/a | Height over experiment |
|  | n/a | Root length over experiment |
| Allocation | SRL | Specific Root Length |
|  | RMR  SLA  LMR  R:S  SMR | Root/Mass Ratio  Specific Leaf Area  Leaf/Mass Ratio  Root/Shoot Ratio  Shoot/Mass Ratio |
| Physiological | LCP  A_max_  R_d_ | Light Compensation Point  Maximum Photosynthetic Rate  Dark Respiration Rate |

**Table S2.** Results of pairwise contrasts of height growth model between AC and EC treatments over the course of the experiment.

| **Time period** | **Estimate** | **SE** | **d.f.** | **t ratio** | **p-value** |
| --- | --- | --- | --- | --- | --- |
| **1** | 0.200 | 0.120 | 124.91 | 1.673 | 0.097 |
| **2** | 0.028 | 0.120 | 125.98 | 0.237 | 0.813 |
| **3** | 0.051 | 0.119 | 122.42 | 0.423 | 0.673 |
| **4** | -0.166 | 0.118 | 120.07 | -1.404 | 0.163 |
| **5** | -0.153 | 0.118 | 120.07 | -1.297 | 0.197 |
| **6** | -0.155 | 0.118 | 120.07 | -1.317 | 0.190 |
| **7** | -0.111 | 0.118 | 120.07 | -0.941 | 0.349 |
| **8** | -0.172 | 0.118 | 120.07 | -1.460 | 0.147 |
| **9** | -0.112 | 0.125 | 144.55 | -0.894 | 0.373 |


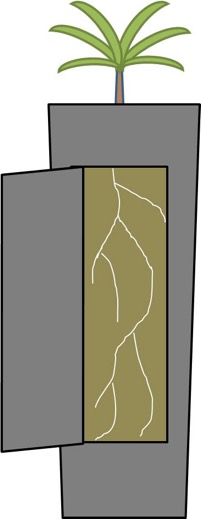


**Figure S1** Schematic of the “windows” created in seedling pots to monitor root growth measurement throughout the experiment. The window was created by inserting a transparency sheet into the pot


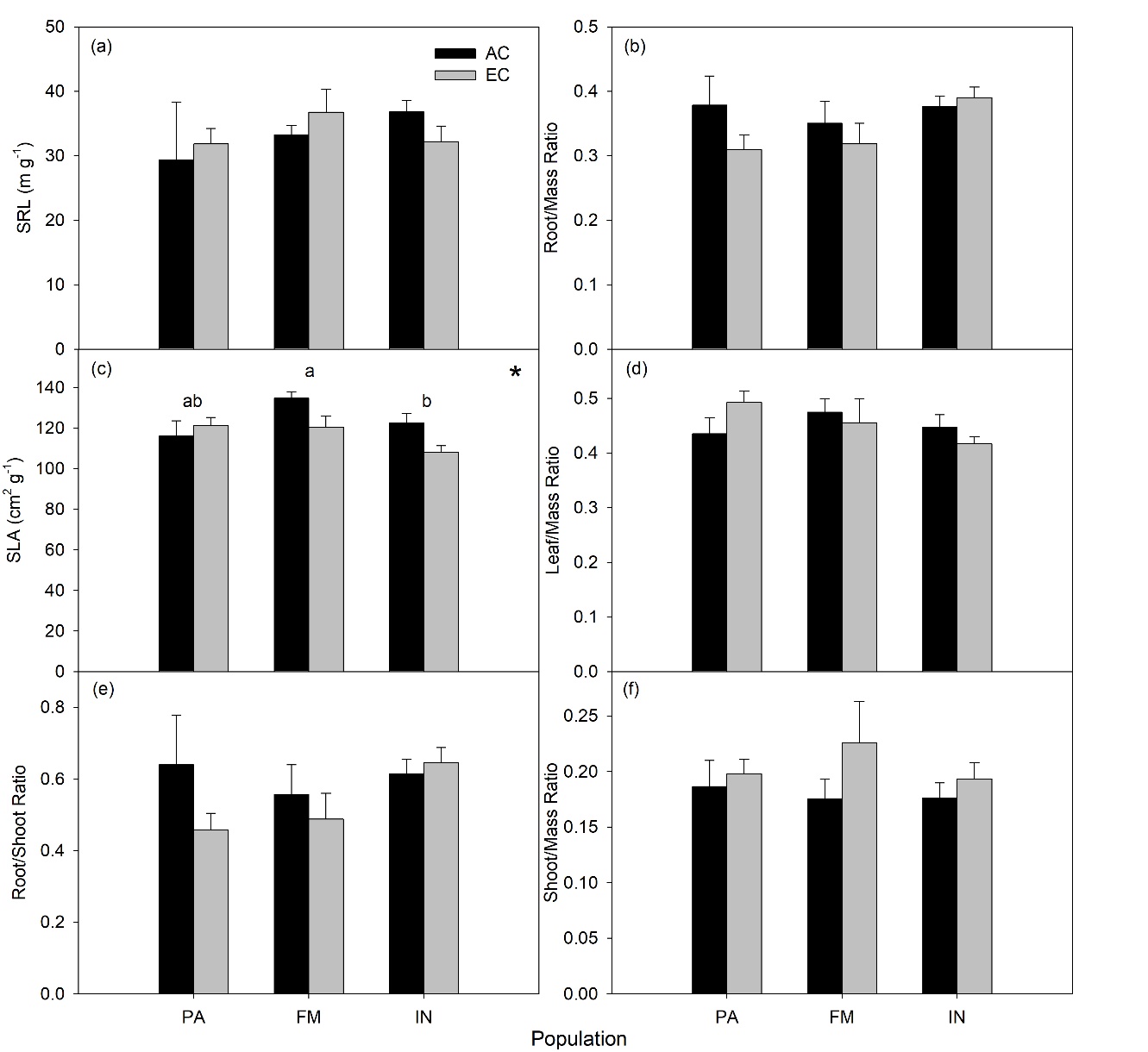


**Figure S2.** Post-harvest allocation trait measurements. Populations shown are Prince Albert SK (PA), Fort McMurray AB (FM), and Inuvik NT (IN) and CO_2_ treatments are ambient CO_2_ (AC) and elevated CO_2_ (EC). Error bars indicate standard error around the means. Significant differences between populations are indicated by letters. Significant differences between CO_2_ treatments are denoted by an asterisk (*) in the upper right corner of the plot.


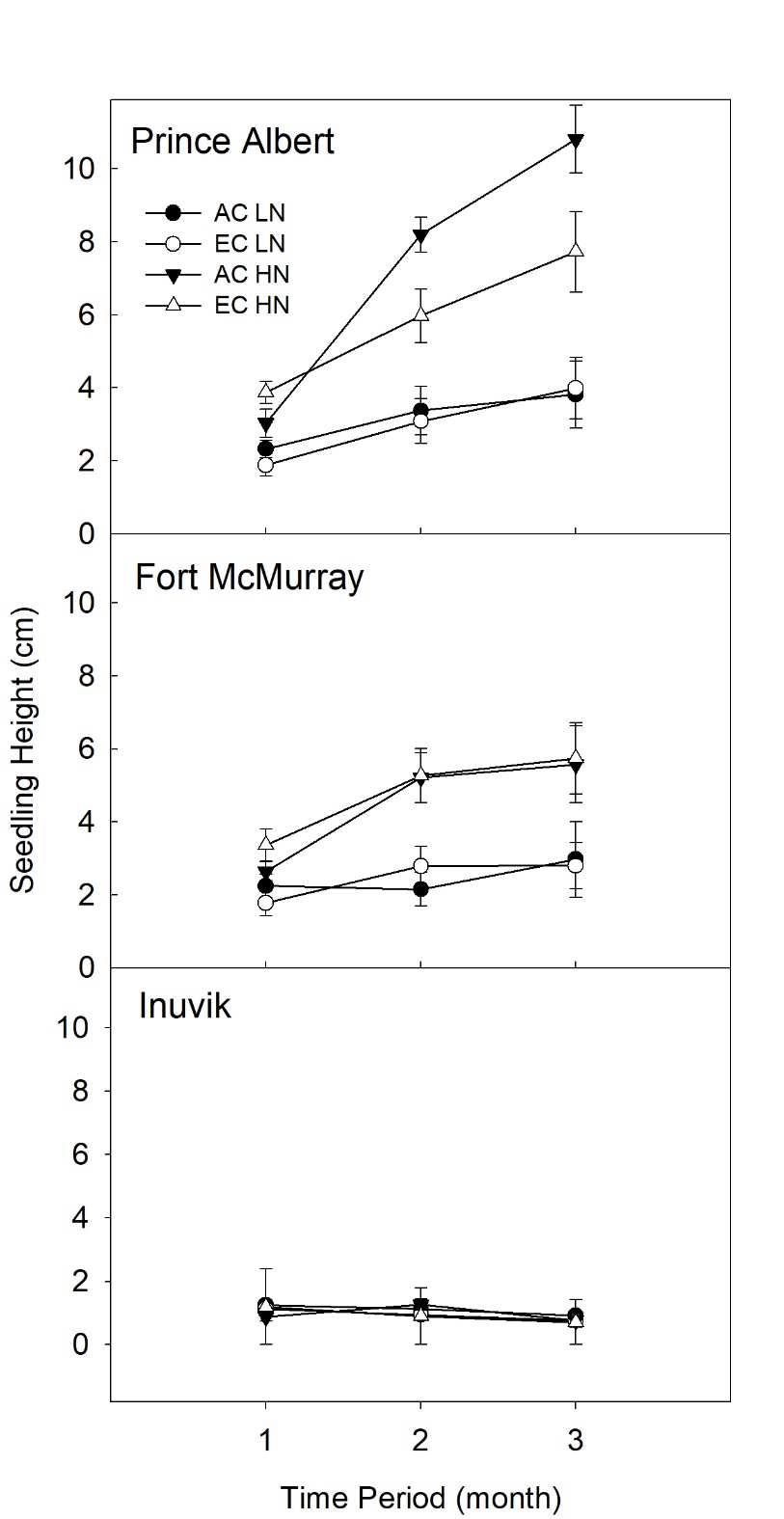


**Figure S3.** Mean seedling height over the first three-month-long growing season. Populations shown are Prince Albert SK (PA), Fort McMurray AB (FM), and Inuvik NT (IN). Ambient CO_2_ treatment is represented by filled symbols (AC), while elevated CO_2_ is shown as unfilled symbols (EC). Error bars represent standard error.
